# Supplementary material for: The causal role of circulating inflammatory markers in osteoporosis: a bidirectional Mendelian randomized study
Source: Front Immunol. 2024 Jul 18;15:1412298. doi: 10.3389/fimmu.2024.1412298 (PMC11291241; doi:10.3389/fimmu.2024.1412298)
Supplement: Supplementary file 1 [file DataSheet_1.zip › supplementary material/OSTEOPOROSIS-CIM/GCST90274779.tsv.gz_forest.pdf]

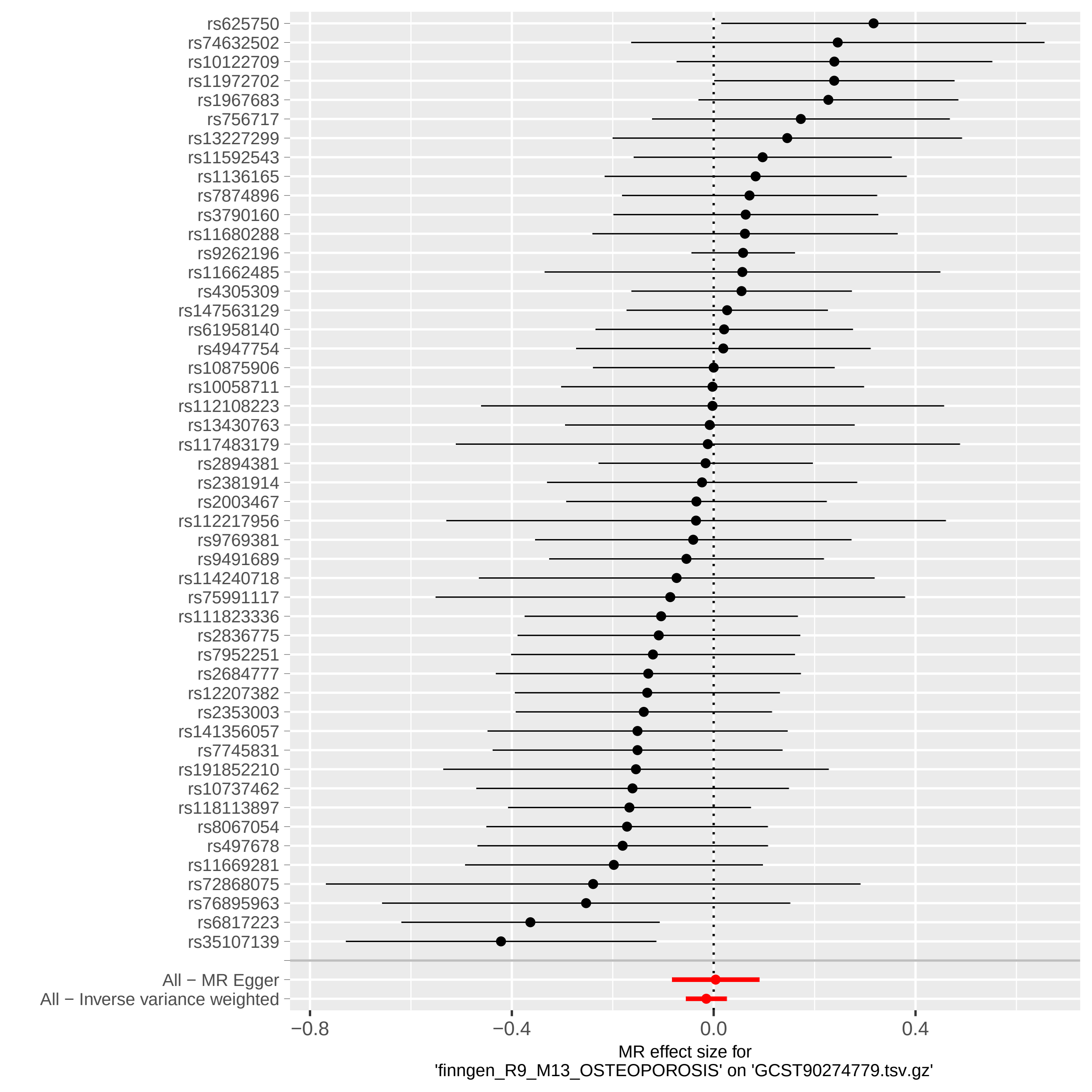
rs625750
rs74632502
rs10122709
rs11972702
rs1967683
rs756717
rs13227299
rs11592543
rs1136165
rs7874896
rs3790160
rs11680288
rs9262196
rs11662485
rs4305309
rs147563129
rs61958140
rs4947754
rs10875906
rs10058711
rs112108223
rs13430763
rs117483179
rs2894381
rs2381914
rs2003467
rs112217956
rs9769381
rs9491689
rs114240718
rs75991117
rs111823336
rs2836775
rs7952251
rs2684777
rs12207382
rs2353003
rs141356057
rs7745831
rs191852210
rs10737462
rs118113897
rs8067054
rs497678
rs11669281
rs72868075
rs76895963
rs6817223
rs35107139
All − MR Egger
All − Inverse variance weighted
−0.8
−0.4
0.0
0.4
MR effect size for
'finngen_R9_M13_OSTEOPOROSIS' on 'GCST90274779.tsv.gz'
